# Abnormal Pressure Pain, Touch Sensitivity, Proprioception, and Manual Dexterity in Children with Autism Spectrum Disorders

**DOI:** 10.1155/2016/1723401

**Published:** 2016-01-05

**Authors:** Inmaculada Riquelme, Samar M. Hatem, Pedro Montoya

**Affiliations:** ^1^University Institute of Health Sciences Research (IUNICS-IdISPa), University of the Balearic Islands, 07122 Palma de Mallorca, Spain; ^2^Department of Nursing and Physiotherapy, University of the Balearic Islands, 07122 Palma de Mallorca, Spain; ^3^Clinic of Physical Medicine and Rehabilitation, Brugmann University Hospital, 1020 Brussels, Belgium; ^4^Institute of Neuroscience, Université catholique de Louvain, Brussels, Belgium

## Abstract

Children with autism spectrum disorders (ASD) often display an abnormal reactivity to tactile stimuli, altered pain perception, and lower motor skills than healthy children. Nevertheless, these motor and sensory deficits have been mostly assessed by using clinical observation and self-report questionnaires. The present study aims to explore somatosensory and motor function in children with ASD by using standardized and objective testing procedures. *Methods*. Tactile and pressure pain thresholds in hands and lips, stereognosis, proprioception, and fine motor performance of the upper limbs were assessed in high-functioning children with ASD (*n* = 27) and compared with typically developing peers (*n* = 30).  *Results*. Children with ASD showed increased pain sensitivity, increased touch sensitivity in C-tactile afferents innervated areas, and diminished fine motor performance and proprioception compared to healthy children. No group differences were observed for stereognosis. *Conclusion*. Increased pain sensitivity and increased touch sensitivity in areas classically related to affective touch (C-tactile afferents innervated areas) may explain typical avoiding behaviors associated with hypersensitivity. Both sensory and motor impairments should be assessed and treated in children with ASD.

## 1. Introduction

Autism spectrum disorders (ASD) have been repeatedly associated with motor and somatosensory impairments. Motor performance is narrowly related to the correct integration of touch sensitivity, as it is shown by the coactivation of brain somatosensory and motor areas during motor tasks [1]. Thus, praxis performance requires representations of the body, movement and environment (mediated by parietal regions), and transcoding of these representations into movement plans (mediated by premotor circuits) [2]. Moreover, sensitivity and motor impairments have been related to the ability to participate successfully in daily life activities in children with ASD [3, 4].

It has been shown that perceptual-motor action models combining somatosensory and motor circuits and necessary to development of skilled gestures, such as manual dexterity, are altered in children with ASD [4]. The development of gross and fine motor function appears to be delayed in children with ASD [5–8] and individuals with ASD exhibited dysfunctional posture and muscle tone, fine manipulative apraxia, lower grip strength, stiffer gait, lack of coordination, lower movement speed, excessive associated movements, and, in general, deficits in planning and execution of motor actions compared to typically developing peers [8–16].

Children with ASD also are characterized by abnormal sensitivity to touch, proprioceptive, and painful stimuli [17, 18]. Thus, previous work studies have found that high-functioning children with ASD self-reported strong reactions and heightened apprehension to external tactile stimuli (hypersensitivity), as well as hyposensitivity to proprioception and pain stimuli [19]. Furthermore, questionnaire data from parents and health professionals have revealed that individuals with ASD displayed substantial alterations on somatosensory perception, varying from hyper- to hyporesponsivity to the same stimulus [20–24] and in different situational contexts [25–27]. In addition, it has been suggested that the apparent reduction of pain reactivity in children with ASD could be due to differences of pain expression related to difficulties with verbal communication, body representation, and cognitive disorders rather than to real analgesia [28, 29].

Furthermore, it has been recently discussed that processing of nonpainful tactile processing stimuli is a complex phenomenon, including characterization of external stimuli (sensory-discriminative dimension), such as in object manipulation, and integration of affective and social information (affective-motivational dimension) [30]. According to recent evidence, detailed information on affective touch and pressure pain would provide relevant clues on the possible causes for behavioral hyperreactivity to bodily stimuli and related avoidance behaviors reported in individuals with ASD from early ages [19, 22]. Nevertheless, it should be noted that previous studies on somatosensory processing in children with ASD are mainly based on self-reported measures; to date, objective assessments of somatosensory processing in adults with ASD have provided only contradictory results. For instance, several studies have found that ASD and healthy adults displayed similar proprioceptive [19, 31, 32], vibrotactile [33], tactile, and thermal thresholds [27], texture discrimination [34], spatial localization on the skin [35], and stereognosis [36]. By contrast, other studies have described higher [37] or lower vibration thresholds [27, 38], lower cold and heat pain thresholds [27], and impaired stereognosis [9] in adults with ASD as compared with healthy controls.

The present study specifically aimed to characterize somatosensory and motor function in children diagnosed with ASD by using standardized psychophysical methods and motor assessments. For this purpose, tactile and pressure pain thresholds, stereognosis, proprioception, and fine motor skills were assessed in a group of children with ASD, compared to typically developing peers. Based on previous research, we hypothesize that children with ASD will have sensoriomotor deficits in variables related to the affective-emotional dimension of touch processing (i.e., pain sensitivity) but not with variables related to the sensory-discriminative dimension (i.e., stereognosis).

## 2. Materials and Methods

### 2.1. Participants

Participants with diagnosis of high-functioning ASD according to DMS criteria [39], reported in their medical history by their neurologist, were recruited from a summer school in Majorca (Spain) in July and August of 2012. Potential participants were identified by their own physicians and invited to participate in a meeting with their parents, where they received detailed information about the experimental protocol. Inclusion criteria were (1) children between 4 and 15 years of age and (2) a cognitive level allowing to understand and to follow simple instructions (e.g., to answer if they felt touch or pain upon stimulation). Age-matched typically developing children, with nondiagnosis of ASD or other developmental disorders, were also recruited from other summer schools during the same time period. All participants were right-handed.

Twenty-seven children with ASD (7 girls; 6.3 yrs ± 3.23) and 30 typically developing peers (15 girls; 6.5 yrs ± 3.37) met the inclusion criteria and agreed to participate in the study. At the time of the study, none of the participants was receiving any physical or occupational therapy. The descritive characteristics of the children with ASD are displayed in Table 1.

Parents or legal tutors signed informed consents and participants gave their oral approval to participate in the study. None of the children/parents withdrew consent or chose to discontinue the study. The study protocol was approved by the Ethics Committee of the Regional Government of the Balearic Islands.

### 2.2. Somatosensory Assessment

Participants were assessed individually by an experienced investigator (IR). Pressure pain and tactile thresholds were determined bilaterally on hands and face. Stereognosis and proprioception were tested on both hands. Participants performed two tests of fine manual dexterity. Testing order of somatosensory stimuli and motor evaluations was randomized. The total duration of the individual assessment was thirty minutes.

#### 2.2.1. Pressure Pain Thresholds

Pressure pain thresholds (expressed in kg/cm^2^) were measured with a digital dynamometer using a flat rubber tip (surface of the tip: 1 cm^2^). Participants were asked to say “pain” or to raise a hand when the pressure became painful and this was considered the pressure pain threshold. Pressure was released when the pain threshold or maximally exerted pressure of the dynamometer was reached. Pressure stimuli were applied pseudorandomly on twelve bilateral body locations: lips, cheeks, thenar eminences, thumb pads, index finger pads, and hand dorsi. Three stimuli were applied at each body location. The average of the three stimuli was calculated as the pressure pain thresholds for each body location. Grand-averages were computed for body locations of FACE (lips and cheeks) and HAND PALM (thenar eminences, thumbs, and index fingers). To avoid anxiety, at the start of the experimental session children were familiarized with the assessment procedure by using several nonpainful stimuli in the same body locations. All children correctly understood and pursued the procedure and any participant expressed distress during its execution. Nevertheless, to avoid any bias due to noncommunication of pain by children with ASD, the child's teachers observed him/her during the procedure to report when signs of distress would appear what would stop the procedure. *Z*-scores were computed to standardize threshold values. The reliability of this procedure for assessing pressure pain sensitivity has been demonstrated in previous studies [40]. The reliability of the capacity to express pain by children with mild cognitive deficits has been shown in previous studies [18, 41, 42].

#### 2.2.2. Tactile Thresholds

Punctate tactile sensitivity was measured with Von Frey monofilaments [43] with a diameter ranging from 0.14 to 1.01 mm according to the method of limits [44] at the same twelve body locations as pressure pain thresholds (see the above). The assessment was performed by touching the skin in a perpendicular way, pressing the monofilament slowly down till it buckled, holding it steady during 1.5 s, and removing it in the same way as it was applied. After several practice trials, children were instructed to express if they felt any touch sensation by saying “yes” or “no.” Null stimuli were applied to check for false positive responses. Responses with more than 3 s delay were considered as undetected. Body locations were stimulated in a pseudorandomized order. The procedure started with a thick filament and depending on the participant's detection, subsequent monofilaments were applied with increasing or decreasing diameters. The tactile detection threshold of each body location was determined as the thinnest filament identified by the participant in three subsequent assessments. Grand-averages were computed for body sites of FACE (lips and cheeks) and HAND PALM (thenar eminences, thumbs, and index fingers). The logarithm of these values was computed. The reliability of the capacity to express tactile sensations by children with mild cognitive deficits has been shown previously [41, 42].

#### 2.2.3. Stereognosis

The ability to perceive and recognize the form of objects by only using tactile information was assessed separately in both hands by using ten common objects (coin, bank note, scissors, pencil, pen, comb, towel, sponge, glass, and cup). Participants wore a sleeping mask and were instructed to touch the object with the hand and to identify it. Stereognosis was scored from 0 to 2 for each object (2 = normal, the object was correctly identified; 1 = impaired, participant was able to describe some features of the object; 0 = absent, participant was unable to identify the object) and a sum score of all ten objects was computed. This procedure was adapted from the Nottingham Sensory Assessment test, whose reliability has been proven in previous studies [45].

#### 2.2.4. Proprioceptive Tasks

The sense of the relative position and movement of several parts of the upper arm was assessed as the ability to reproduce passive joint movements (wrist, elbow, metacarpophalangeal joints from the second to the fifth digit, and metacarpophalangeal joint of thumb) performed by the experimenter when participants were wearing a sleeping mask. Proprioception was scored according to following criteria: 2 = normal, able to achieve final joint position within 10° range of error; 1 = partially impaired, able to appreciate joint movement but failed to detect movement direction; 0 = impaired, no appreciation of joint movement. This procedure was adapted from the Nottingham Sensory Assessment test, whose reliability has been proven in previous studies [45].

### 2.3. Fine Motor Skills

The Purdue Pegboard test was used to assess fine finger dexterity. During the test, the child was seated in front of a pegboard with two cups containing 25 pins and located at the far-right and far-left corner. The task consisted in picking up one pin at a time from each cup by using the thumb and index finger only and placing it in the appropriate row (left or right). Children were instructed to place as many pins as possible in 30 s. Two trials were performed: one with the right hand and one with the left hand. The number of correctly inserted pins was used as test score. The testing procedure with both hands and the assembly part of the original test were not used in this research protocol. The Purdue pegboard test has been used successfully to assess fine hand performance in children with motor disabilities [46].

The Box and Block test was used to assess gross manual dexterity. Both hands were tested separately. The child was seated in front of a table facing a rectangular box divided into two equal compartments by a 15.2 cm high partition panel. Children were instructed to transfer as many cubes (2.5 cm^3^) as possible, one at a time, from one compartment to another in one minute. Only trials in which the child's hand crossed over the partition line were considered as correctly executed. Blocks that dropped out from the second compartment onto the floor were scored as correct. Those trials in which several blocks were transferred at the same time were scored as one cube transfer. The total number of correctly transferred cubes with each hand was computed. This test has been used previously to assess gross manual dexterity in individuals with ASD [47].

### 2.4. Statistical Analyses

As normality test showed no significant differences (all Kolmogorov-Smirnoff *Z* < 1.20, all *P* > .093), analyses of variance (ANOVA) were used to test the interactions of between-subject factors GROUP (children with ASD versus typical developing peers) and GENDER (boys versus girls) and the within-subjects factor BODY SIDE (right versus left). An additional within-subjects factor BODY LOCATION (face versus hand dorsum versus hand palm) was used to analyze touch and pressure pain thresholds. ANOVA results were adjusted by using Bonferroni corrections for post hoc comparisons and Greenhouse-Geisser corrections for the violation of sphericity assumptions. Pearson correlations were performed to determine the influence of age in the different tests.

## 3. Results

### 3.1. Pressure Pain Thresholds

**Figure 1:**
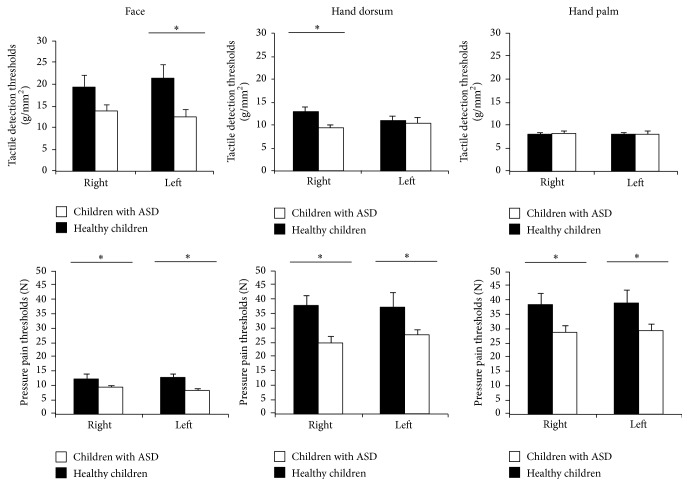
Tactile detection and pressure pain thresholds in face, hand palm, and dorsum for each group (children with ASD versus healthy children), separated by body locations (face versus hand palm versus hand dorsum) and body side (right versus left). Pressure pain thresholds were significantly lower in ASD children, whereas tactile detection thresholds were similar to healthy controls in hand palm but significantly lower on face and hand dorsum. Results are displayed as mean ± SD. ANOVA: ^*∗*^ *P* < .05, ^*∗∗*^ *P* < .01.

A significant main GROUP effect was found on pressure pain thresholds (*F*(1,46) = 4.08, *P* = .049), showing lower thresholds in children with ASD (mean *Z*-score = −.26, SD = .22) than in their typically developing peers (mean *Z*-score = .32 SD = .19) (Figure 1).

### 3.2. Tactile Thresholds

Significant effects due to BODY LOCATION (*F*(2,47) = 367.84, *P* < .001) (face mean *Z*-score = 1.14, SD = .03; hand dorsum mean *Z*-score = 2.29, SD = .05; hand palm mean *Z*-score = 2.04, SD = .04) and GROUP × BODY LOCATION × BODY SIDE were found for tactile thresholds (*F*(2,47) = 4.83, *P* = .028) (Figure 1). Post hoc comparisons indicated that typically developing children had significant higher tactile thresholds than children with ASD in left face and right hand dorsum (dominant hand) (both *P* < .037). Moreover, the three body locations were significantly different in typically developing children (face < hand palm < hand dorsum) (*P* < .001); whereas significant differences were only observed between face and hand palm (face < hand palm) (*P* < .001) and face and hand dorsum (face < hand dorsum) (*P* < .001) in children with ASD. Tactile thresholds were higher at the left (nondominant hand) than at the right hand dorsum (dominant hand) (*P* < .024) in typically developing children, whereas there were no differences due to BODY SIDE in children with ASD.

### 3.3. Stereognosis

**Figure 2:**
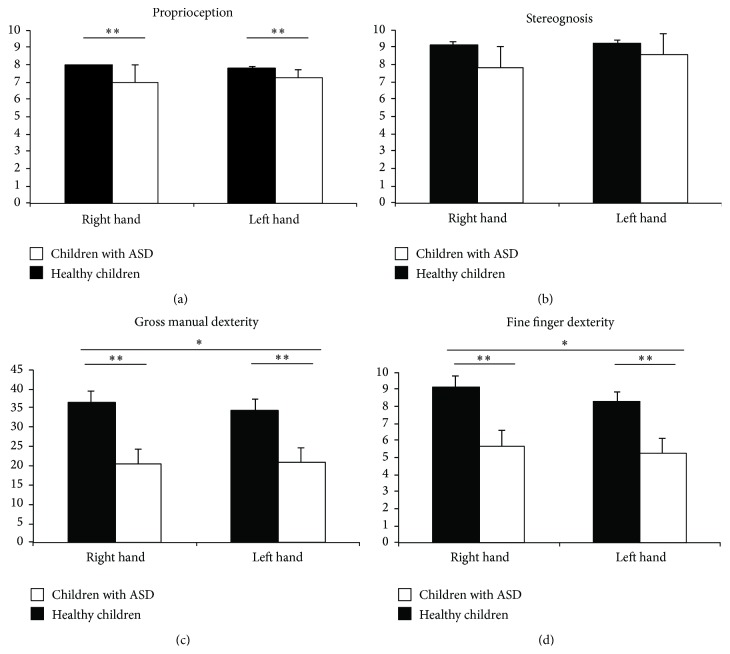
Stereognosis, proprioception, gross manual dexterity, and fine finger dexterity scores for each group (children with ASD versus healthy children) separated by body side (right versus left). Stereognosis was normal in ASD children. Proprioception, gross, and fine manual dexterity were significantly impaired compared to healthy children. Results are displayed as mean ± SD. ANOVA: ^*∗*^ *P* < .05, ^*∗∗*^ *P* < .01.

Behavioral performance on stereognosis tests did not differ between groups (*P* > .422). The percentage of correct trials was 92% for typically developing children and 85% for children with ASD (Figure 2(a): proprioception).

### 3.4. Proprioceptive Tasks

Significant group differences were found for proprioception measurements (*F*(1,31) = 7.31, *P* = .011), showing decreased proprioception scores in children with ASD (mean = 7.13 (maximum score = 8), SD = .27) compared with typically developing children (mean = 7.90 (maximum score = 8), SD = .10) (Figure 2(b): stereognosis).

### 3.5. Fine Motor Skills

Gross and fine manual dexterity was reduced significantly in children with ASD. Significant main effects due to the factor GROUP were found in both gross manual (*F*(1,44) = 8.42, *P* = .006) and fine finger dexterity (*F*(1,44) = 9.61, *P* = .003), revealing decreased manipulative dexterity in children with ASD (gross manual dexterity: mean = 20.97, SD = 3.99; fine finger dexterity: mean = 5.44, SD = .85) compared with typically developing children (gross manual dexterity: mean = 35.32, SD = 2.92; fine finger dexterity: mean = 8.70, SD = .62). Also, significant differences due to BODY SIDE were found in gross manual (*F*(1,44) = 4.09, *P* = .049) and fine finger dexterity (*F*(1,44) = 5.31, *P* = .026), revealing that all children were more skilled with the dominant hand (i.e., right hand) (gross manual dexterity: mean = 28.81, SD = 2.50; fine finger dexterity: mean = 7.38, SD = .57) than with the nondominant (left) hand (gross manual dexterity: mean = 27.47, SD = 2.49; fine finger dexterity: mean = 6.76, SD = .51) (Figures 2(c) and 2(d)).

No significant effects were found for the main factor GENDER or any of their interactions in any of the variables.

Age showed significant positive correlations with pressure pain thresholds of all the areas in the typically developing children (all *r* > .577, all *P* < .01) indicating a decreasing of pain sensitivity with age; in contrast, children with ASD only showed significant positive correlations with pain in palms (all *r* > .531, all *P* < .013) and in left face (*r* = .829, *P* < .001). Although typically developing children showed significant positive correlation between age and stereognosis (all *r* > .401, all *P* < .014) indicating an improvement of stereognosis with age, no significant correlations were found in children with ASD. Finally, age showed significant positive correlations in all dexterity tests in all the children (typically developing children: all *r* > .84, all *P* < .001; children with ASD: all *r* > .52, all *P* < .040), indicating better motor performance with development. No significant correlations were found between age and tactile thresholds or proprioception for any of the groups.

## 4. Discussion

The aim of the present study was to assess somatosensory function in face and hands and motor function of the upper limbs in children with ASD in comparison with typically developing children. Children with ASD displayed lower pressure pain thresholds (higher pain sensitivity) than their typically developing peers. Also, children with ASD displayed higher tactile sensitivity at the face and hand dorsum than typically developing children. Interestingly, children with ASD displayed no significant differences between hand palm and hand dorsum tactile thresholds, as it was found in typically developing children. We also observed that children with ASD were less skilled in object manipulation and had poorer upper limb proprioception than their typically developing peers; by contrast, no differences were found on stereognosis. These effects seem to be gender-independent but differently related to age development in both groups.

The present findings in children with ASD showed tactile and pain hypersensitivity assessed by objective neuropsychological test, in contrast with the conflicting evidence provided by studies based on questionnaires [19–24]. Our results are in accordance with previous studies on adults with ASD that used similar tests, indicating increased sensitivity to thermal pain [27] and normal stereognosis compared to healthy adults [36]. The relevance of the present data is stressed by the negative influence of tactile hypersensitivity in individuals with ASD on social behaviors that involve interpersonal touch [17, 48]. The lack of body-location-related differentiation of touch sensitivity (as reported in children with ASD in the present study) may suggest major alterations of somatosensory processing. In healthy subjects, neuroimaging and electrophysiological data have shown that discriminative and affective components of pleasant touch are mediated by different tactile mechanoreceptors fibers and may be differentially correlated with the activation of specific brain areas involved in somatosensory processing [30, 49]. Thus, for instance, it has been found that pleasant touch from hairy skin is associated with the peripheral activation of unmyelinated C fibers and leads to activations of posterior insular cortex and midanterior orbitofrontal cortex, whereas similar touch on glabrous skin may be signaled by A-beta afferents and elicits activations of somatosensory cortices [8, 50–52]. Moreover, unmyelinated C fibers afferents have been considered as prime candidates for tactile hypersensitivity associated with ASD disorders [27, 53]. Thus, Kaiser et al. [53] reported different brain activation responses in structures of the socioemotional network and the somatosensory cortex depending on the tactile stimulation of CT-fibers or non-CT innervated areas in children and adolescents with ASD. The relationship between peripheral C-fibers and the affective component of somatosensory perception readily could explain why tactile elicits abnormally low responses in face and hand dorsum but not in hand palm, in our study population of children with ASD. Our present results suggest that the perceptual phenomenon of altered tactile sensitivity in persons with ASD could be attributable to an alteration in affective touch processing rather than to an impaired detection of tactile stimuli [33, 48]. Although Cascio et al. [27] did not find hypersensitivity in Von Frey touch thresholds in adults with ASD, they reported an increased sensitivity for detection of vibration in C-innervated area (forearm) but not in A-beta innervated area (palm), which would be in accordance with our results and would support the hypothesis of an alteration in affective touch processing in persons with ASD.

The higher pain sensitivity observed in our children with ADS also could be due to an abnormal processing of the affective component of pain. Pressure pain or blunt pain perception, as with the pressure stimuli used in our study, is mediated by C-fibers [54]. Moreover, children with ASD experienced an age-related pain sensitivity only in glabrous areas. In support of the hypothesis that abnormal processing of the affective-motivational dimension of touch, which would integrate affective and social touch information, may be the cause of adverse reactions to touch, the present results clearly show that tactile and pain thresholds in children with ASD are impaired. Children with ASD in the present study were rather insensitive than hypersensitive to tactile stimuli that did not contain an affective component.

The results of the present study further revealed impaired fine motor performance in children with ASD, although it had a similar development pattern, compared to typically developing children. Other studies also have shown manipulation deficits such as longer execution time in reaching tasks, increase in the duration of unloading, impaired coordination, and reduced grip strength in individuals with ASD [9, 15, 55]. In addition to these motor disabilities, we also found reduced proprioception in children with ASD [19, 31, 32]. Proprioceptive dysfunction has been previously related to poor movement strategies in children with ASD [20] and in children with other pathological conditions, such as cerebral palsy [55] or primary dystonia [56]. Our findings corroborate previous evidence suggesting that children with ASD may suffer from a more general involvement of neural functions beyond those regulating social and communication behavior [8]. In absence of impairments of the sensory-discriminative dimension of touch processing, further research is needed to deepen the influence of affective-emotional dimension of touch processing in motor praxis. Nevertheless, motor skills have been reported as an important predictor of child's performance in daily life activities, such as handwriting and school function [4] and thus should be taken into account when intervening to improve ASD children's autonomy.

The main limitation of the present study is that the study protocol and the somatosensory stimuli used for the evaluation were not appropriate to check for the unpleasantness of stimuli or to specifically measure the affective component of somatosensory afferences. Taking into account that affective aspects may influence somatosensory processing, future studies should include assessment protocols that specifically target the emotional and social context in which tactile hypersensitivity appears to occur in children with ASD [27]. Information about medication was not collected, which might have produced some bias in the results.

## 5. Conclusion

The present study indicated that discriminative touch, pressure pain, and motor function of the upper limbs in children with ASD were significantly altered compared to typically developing children. Both, sensory and motor processing impairments might influence participation of children with ASD in daily activities and must be taken into account for a therapeutic intervention [17]. Sensory-discriminative and affective-motivational brain processing of touch develops throughout infancy [26] and early impairments in somatosensory processing may influence later stages of cortical activity and have consequences along all the life spam [27, 57]. Until now, research on pain and somatosensory hyper/hyposensitivity in children with ASD has relied almost exclusively on observational or behavioral assessment measures. Insights into the neurobiological basis of somatosensory processing may provide a more robust and objective way to investigate pain and tactile sensitivity in individuals with ASD [25]. Further psychophysiological investigation of sensory abilities is required to clarify the roles of sensation, perception, and affect in individuals with ASD. This is specially relevant considering that information from C-tactile afferents in posterior insular cortex provides a basis for encoding caresses, recognizing touch hedonic relevance, and activating key nodes of the “social brain” [30, 58]. Since social relevance of affective touch extends to the touch interactions of others, the abnormal developing of these brain networks may result in disorders related to social processing in children with ASD. Understanding how different brain structures contribute to the abnormal processing of somatosensory stimuli may help to better characterize the relationship between somatosensory perception and behavior in children with ASD and may lay the basis to develop interventions for maximizing social participation in individuals with ASD.

## Figures and Tables

**Table 1:** Descriptive characteristics of the children with autism spectrum disorders.

	Number of children
Gender	
Males	20
Females	7
Age	6.3 years ± 3.23
Cognitive impairment	
None	24
Mild	3
Moderate	0
Severe	0
Verbal ability	
Fluent communicative speech	12
Speech with communicative sentences but frequent echolalia	7
A few communicative sentences	4
A few words	4
Nonverbal expression	0
